# Impact of observability period on the classification of COPD diagnosis timing among Medicare beneficiaries with lung cancer

**DOI:** 10.1371/journal.pdig.0000633

**Published:** 2024-10-22

**Authors:** Eman Metwally, Sarah E. Soppe, Jennifer L. Lund, Sharon Peacock Hinton, Caroline A. Thompson

**Affiliations:** 1 Department of Epidemiology, Gillings School of Global Public Health, The University of North Carolina at Chapel Hill, Chapel Hill, North Carolina, United States of America; 2 Lineberger Comprehensive Cancer Center, The University of North Carolina at Chapel Hill, Chapel Hill, North Carolina, United States of America; 3 Center for Health Promotion and Disease Prevention, The University of North Carolina at Chapel Hill, Chapel Hill, North Carolina, United States of America; Beth Israel Deaconess Medical Center, UNITED STATES OF AMERICA

## Abstract

**Background:**

Investigators often use claims data to estimate the diagnosis timing of chronic conditions. However, misclassification of chronic conditions is common due to variability in healthcare utilization and in claims history across patients.

**Objective:**

We aimed to quantify the effect of various Medicare fee-for-service continuous enrollment period and lookback period (LBP) on misclassification of COPD and sample size.

**Methods:**

A stepwise tutorial to classify COPD, based on its diagnosis timing relative to lung cancer diagnosis using the Surveillance Epidemiology and End Results cancer registry linked to Medicare insurance claims. We used 3 approaches varying the LBP and required continuous enrollment (i.e., observability) period between 1 to 5 years. Patients with lung cancer were classified based on their COPD related healthcare utilization into 3 groups: pre-existing COPD (diagnosis at least 3 months before lung cancer diagnosis), concurrent COPD (diagnosis during the -/+ 3months of lung cancer diagnosis), and non-COPD. Among those with 5 years of continuous enrollment, we estimated the sensitivity of the LBP to ascertain COPD diagnosis as the number of patients with pre-existing COPD using a shorter LBP divided by the number of patients with pre-existing COPD using a longer LBP.

**Results:**

Extending the LBP from 1 to 5 years increased prevalence of pre-existing COPD from ~ 36% to 51%, decreased both concurrent COPD from ~ 34% to 23% and non-COPD from ~ 29% to 25%. There was minimal effect of extending the required continuous enrollment period beyond one year across various LBPs. In those with 5 years of continuous enrollment, sensitivity of COPD classification (95% CI) increased with longer LBP from 70.1% (69.7% to 70.4%) for one-year LBP to 100% for 5-years LBP.

**Conclusion:**

The length of optimum LBP and continuous enrollment period depends on the context of the research question and the data generating mechanisms. Among Medicare beneficiaries, the best approach to identify diagnosis timing of COPD relative to lung cancer diagnosis is to use all available LBP with at least one year of required continuous enrollment.

## Author summary

Healthcare data such as electronic health records and health insurance claims is a rich resource to study outcomes of chronic medical conditions such as chronic obstructive pulmonary disease (COPD). However, it can be tricky to determine the optimal period of searching the claims to identify the first diagnosis time of a chronic condition. In this study, we explored three approaches to optimize classification of COPD diagnosis based on a variety of Medicare claims search periods and observability (required continuous periods of enrollment) periods. We used a large dataset of Medicare claims linked to SEER cancer registry including more than 158,000 Medicare beneficiaries to identify diagnosis timing of COPD relative to lung cancer diagnosis. We observed that the most efficient approach to classify COPD diagnosis timing is to use all available historical claims data while requiring a minimum of one year of Medicare continuous enrollment. Our findings will help researchers concerned about accuracy of diagnosis timing of chronic conditions such as COPD and loss in sample size, especially when long periods of continuous insurance enrollment isn’t feasible.

## Introduction

Investigators often want to use administrative healthcare data to estimate the timing of initial diagnoses of chronic conditions to examine its association with health outcomes. However, healthcare utilization (HCU) data (electronic health records (EHRs) and administrative claims) can be discontinuous based on several factors such as insurance coverage, healthcare access, and severity of the underlying condition, which could prevent achieving this goal. To estimate the time of first diagnosis of a chronic condition, the current recommendations suggest ensuring at least one or two years of observable lookback period prior to the appearance of the chronic condition diagnosis in the claims [1]. The observability period is frequently approximated by the continuous enrollment period (i.e., membership period in coverage through a particular insurance provider), while the look back period (LBP) is the claim search period for indicators of case definition before the index date (e.g., the baseline date or date of diagnosis of a second condition). The LBP could extend to the beginning of all patient data (“all available” LBP) or it extend for fixed amount of time (“fixed” LBP) [2,3]. When the observable LBP period is too short, under-ascertainment of a chronic condition or misclassification of its first diagnosis date can occur because it is not possible to distinguish between missing data and absence of the condition. Indeed, a short continuous enrollment period might result in missing some patients who don’t have HCU related to the chronic condition within that period, or incorrectly classifying their condition diagnosis as incident instead of prevalent [4,5].

To mitigate concerns of “missing” a condition due to shorter continuous enrollment periods, another approach is to restrict the study to those with long periods of continuous enrollment (e.g., 2 or more years). However, basing the study inclusion criteria on longer period of continuous enrollment can lead to selection bias and limit generalizability of the study results due to inadvertent exclusion of younger populations (especially with age-dependent insurance enrollment such as Medicare), groups of patients who have intermittent access to health insurance, or patients with mild underlying chronic conditions. In addition, restricting the population to patients with longer continuous enrollment might be inefficient due to reductions in sample size which can impact the power of the study to detect an association [6]. Previous research have examined the impact of shorter versus longer continuous enrollment on patients’ classification in pharmacoepidemiology studies [7], as well as “fixed” LBPs versus “all available” LBPs in ascertainment of incident versus prevalent chronic conditions [2] and control of confounders using both real world and simulation data [8,9].

In this paper we illustrate a stepwise tutorial for using administrative claims (Medicare) data to optimally classify a commonly occurring chronic condition, chronic obstructive pulmonary disease (COPD), based on its diagnosis timing relative to lung cancer diagnosis. Our objectives are to identify whether each patient with lung cancer has COPD, and if yes, whether COPD diagnosis was prevalent (pre-existing) versus incident (concurrent) at the time of lung cancer diagnosis. We consider how parsing through various periods of lookback and continuous enrollment (observability) can impact classification of COPD diagnosis timing, while considering the sample size and generalizability of the study results.

### Motivating example

COPD is a common comorbidity and an important determinant of lung cancer outcomes [10,11]. However, COPD is frequently underdiagnosed in the general population, and is sometimes not identified until a patient presents with another lung-related condition, such as lung cancer [12,13]. We conducted a study to estimate prevalence versus incidence of COPD diagnosis among a cohort of Medicare beneficiaries with lung cancer, with consideration of when COPD was first diagnosed relative to incident lung cancer diagnosis. Because healthcare utilization of COPD varies with disease severity, access to healthcare, underlying comorbidities and frailty, the correct LBP and continuous enrollment period for establishing a classification of COPD were not immediately clear. Therefore, we varied the LBP and the required continuous enrollment period from one to five years to examine the impact of longer vs. shorter periods on classification of COPD diagnostic timing. We also demonstrated the impact of requiring longer versus shorter periods of continuous enrollment on sample size, the sociodemographic and clinical composition of the population under study.

## Methods

### Data source

We used the National Cancer Institute’s Surveillance, Epidemiology and End Results (SEER) cancer registry data linked to Medicare enrollment and insurance claims data from the Centers for Medicaid and Medicare Services (CMS). The SEER-Medicare data reflect linkage of two large population-based sources to provide detailed information about Medicare beneficiaries with cancer. The SEER database includes data from 21 cancer registries and represents approximately 40% of the US population [14]. The SEER registry file includes clinical, sociodemographic and cause of death information for patients with cancer. The Medicare fee-for-service (FFS) claims files includes billed health care services that occurred in different settings (e.g., hospitals, physician offices, outpatient clinics) from the time of a person’s Medicare enrollment until death.

### Study population

**Fig 1:**
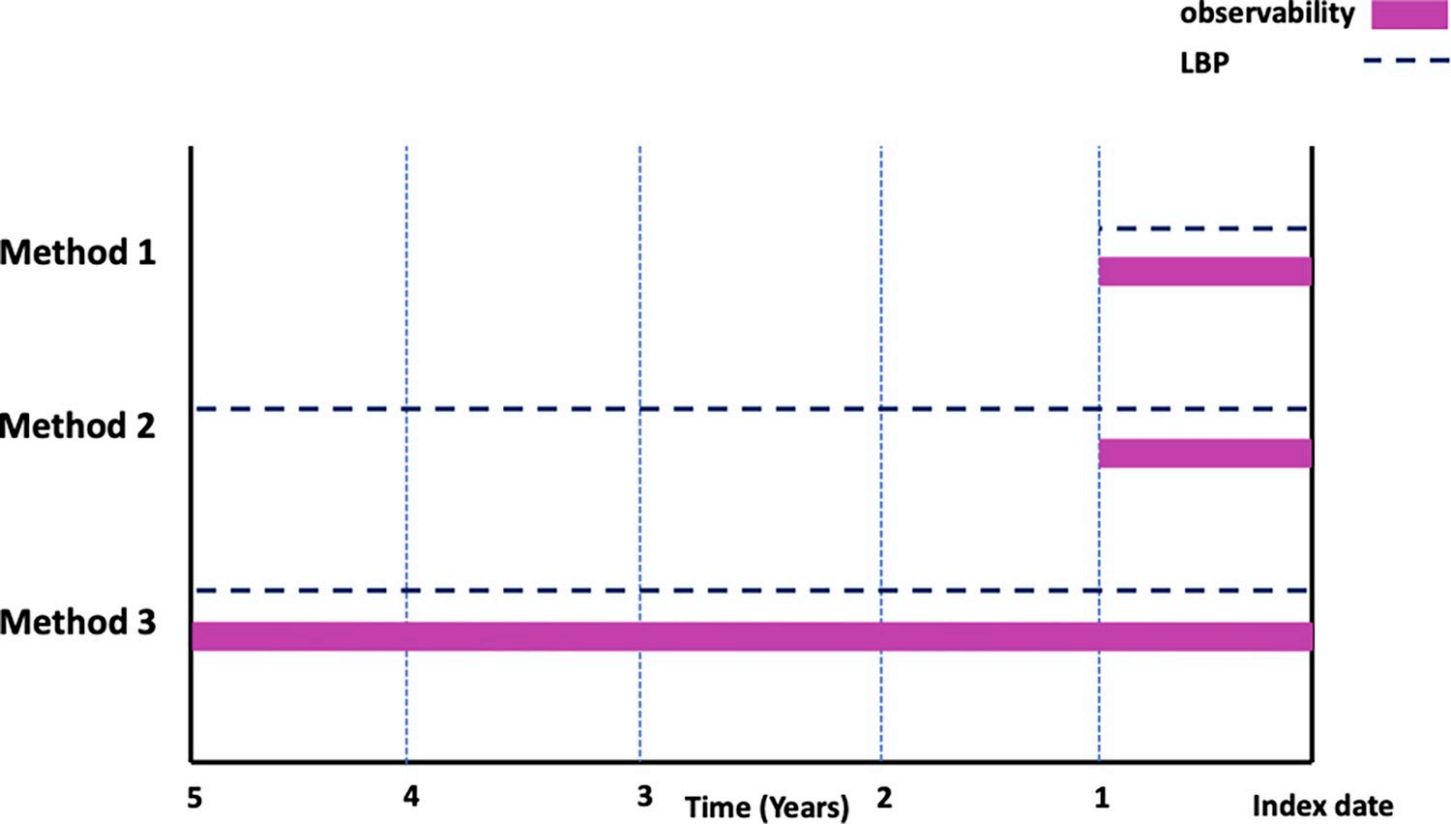
Look back periods (LBPs) and observability (i.e., Medicare fee-for-service continuous enrollment) periods used in Methods 1,2,3 of the study to ascertain COPD diagnosis timing relative to the index date (incident lung cancer diagnosis).

The baseline study population included Medicare beneficiaries who were newly diagnosed with primary invasive lung cancer between 2008 to 2017. We excluded patients who were diagnosed at autopsy or only on their death certificate, had lung cancer staged in situ, had previous history of cancer, or were younger than 66 years old (**Fig 1**). For the baseline analysis, we included patients who had continuous enrollment in parts A and B fee for service without health maintenance organization (HMO) coverage for at least 12 months before to 3 months after lung cancer diagnosis (or until death). For exploration, we identified 4 subpopulations of the baseline population with longer continuous enrollment periods of 2, 3, 4, and 5 years prior to lung cancer diagnosis. To ensure that all study participants were representative of the Medicare population (and the older adult population with lung cancer in the US), we excluded patients younger than 67, 68, 69, and 70 years of age for the subpopulations with required longer continuous enrollment periods of 2, 3, 4, and 5 years, respectively.

Since assumptions made about the data are important to interpret the study approach and results [15], we explicitly state ours: we assumed that Medicare claims is a valid source of data for ascertainment of COPD diagnosis based on its related healthcare utilization; and that once COPD is diagnosed, the patient will live with COPD until lung cancer diagnosis or until death. We considered the longest LBP to be 5 years before lung cancer diagnosis to minimize missing data among patients younger than 70 years old.

### Exposure

To identify patients with comorbid COPD, we used a previously validated algorithm of ICD codes against pulmonologists chart review for pulmonary function testing and other clinical indicators of COPD diagnosis (sensitivity = 85%, specificity = 78.4%) [16,17]. Following this algorithm, we considered COPD diagnosis with one or more of the following ICD diagnosis codes: ICD-9 (491, 491.2, 492, 496) or ICD-10 (J40, J41, J43.0, J43.1, J43.2, J43.8, J43.9, J44). We classified COPD based on its related healthcare utilization in Medicare claims into: **pre-existing COPD**, defined as having the first COPD diagnostic code identified at least 3 months before lung cancer diagnosis, and **concurrent COPD**, defined as having the first COPD diagnostic code +/-3 months from the lung cancer diagnosis.

### Covariates

We described the patient populations by their sociodemographic characteristics, including age at lung cancer diagnosis, sex, race and ethnicity, marital status, census tract-based estimates of socioeconomic status (Yost US-based quintile) [18], residence [19], SEER registry region, and year of lung cancer diagnosis (2008–2017).

### Approaches to classify COPD diagnosis timing relative to lung cancer diagnosis

We used three methods to estimate diagnosis timing of initial COPD relative to lung cancer diagnosis in our study population:

**Method 1: COPD Classification based on a fixed one-year LBP for the baseline cohort with at least one-year continuous enrollment:** Our primary classification of COPD diagnosis timing was based on a one-year LBP searching the claims for evidence of COPD-related HCU among patients with at least one year of continuous enrollment. If the earliest diagnosis code for COPD occurred at least 3 months prior to lung cancer diagnosis, we considered the patient to have pre-existing COPD diagnosis, while if it occurred during the lung cancer peri-diagnosis period (-3 months to +3 months from cancer diagnosis) [20] we considered the patient to have concurrent COPD. Given that some patients may visit their doctors less than once per year, this one-year LBP might not be sufficient to capture the true prevalence of pre-existing COPD. Therefore, we decided to extend the LBP beyond one year to identify additional claims-based evidence of COPD diagnosis that may have occurred earlier (Method 2).

**Method 2: COPD classification based on all available LBPs (truncated at 5 years) for the baseline cohort with at least one year of continuous enrollment.** Using the same baseline cohort (with required one year of continuous enrollment as Method 1), we extended the LBP to 2, 3, 4, and 5 years before lung cancer diagnosis to identify additional evidence of pre-existing COPD. Note that this method is similar to the all-available LBP approach but is truncated at 5 years to avoid missing data among patients younger than 70 years old. In addition, we excluded individuals with early “atypical” Medicare eligibility who are less than 65 years old at the time of enrollment because they are not representative of the older adult population with lung cancer in the US [21]. However, the all-available LBP could introduce misclassification that differentially impacts younger patients whose continuous enrollment period of one year is shorter than the specified LBPs of 2, 3, 4, and 5 years. For example, a patient who is 66 years old at the time of lung cancer diagnosis will not have more than one year of continuous Medicare enrollment because Medicare enrollment begins at 65 years old. Therefore, we tried method 3.

**Method 3: COPD Classification based on 1, 2, 3, 4, and 5 year LBPs for cohorts with at least 5 years of continuous enrollment:** To avoid misclassification of patients with shorter continuous enrollment, we extended the required period of continuous enrollment to match the longer LBPs of 2, 3, 4, and 5 years. Note that Method 3 now introduces the potential for reduction in sample size and generalizability of our study population, especially for younger patients who might not have sufficient enrollment prior to their cancer diagnosis (**Fig 1**).

### Statistical significance of differences between methods

We used Cohen’s Kappa test [22] to compare the agreement between Method 1 and method 2 since they have the same baseline population (n = 185,405). We didn’t compare method 3 with either methods 1 or 2 since it is based on the subpopulation with 5 years continuous enrollment (n = 136,335).

### Sensitivity of various LBPs to classify COPD

We assumed that sensitivity of a LBP to classify COPD will increase if we ensured observability (required continuous enrollment for the entire length of the LBP). Therefore, we measured sensitivity of various LBPs (1, 2, 3, 4 years) against the longest LBP of 5 years (the reference perios) among patients with at least 5 years of Medicare continuous enrollment. Sensitivity of a LBP was calculated as the number of individuals with pre-existing COPD using a shorter LBP divided by the number of individuals with pre-existing COPD using the 5-year LBP (the reference period). The upper and lower limits of 95% confidence interval were calculated based on standard error equations [23] (S1 Text). Because prevalence of pre-existing COPD diagnosis consistently increased with longer LBPs, we set the specificity to 100% across all periods of lookback and continuous enrollment (i.e., no false positive diagnosis of pre-existing COPD).

### Statistical significance of differences between methods

We used Cohen’s Kappa test [22] to calculate the inter-rater agreement (95% CI) between Method 1 and method 2 using the baseline population (n = 185,405). We didn’t use Kappa test to compare method 3 with either methods 1 or 2 because it is based on a subpopulation with smaller sample size (n = 136,338).

All analyses were conducted using SAS, version 9.4 (SAS Institute Inc). This study was reviewed and approved as exempt by The University of North Carolina at Chapel Hill Institutional Review Board (#22–2998).

## Results

The baseline study population included 185,405 older adults with lung cancer with at least one year of continuous Medicare enrollment, of which 70.8% had COPD based on one-year LBP. The mean age at lung cancer diagnosis was 76.4 years old, 50.9% were female, 84.3% were Non-Hispanic White, 7.2% were Non-Hispanic Black, and 4% were Hispanic. Requiring longer continuous enrollment reduced the distribution of patients younger than 70 years old (from 19.3% to 0%), increased prevalence of COPD diagnosis (from 70.8% to 74.6%), without notable changes in other sociodemographic or clinical characteristics. The sample size was reduced by 22.9% from n = 185,405 of the baseline population with one-year continuous enrollment to n = 136,338 of those with at least 5 years continuous enrollment. Most patients in our study (77.1%) had at least 5 years of continuous Medicare enrollment. (**Table 1**).

**Table 1:** Comparing Sociodemographic and clinical characteristics of the study subpopulations based on length of Medicare fee-for-service continuous enrollment.

Characteristics	One-year continuous enrollment N (Col%)	Two-year continuous enrollment N (Col%)	Three-year continuous enrollment N (Col%)	Four-year continuous enrollment N (Col%)	Five-year continuous enrollment N (Col%)
**All patients, N (%)**	185,405 (100)	172,042 (100)	159,542 (100)	147,674 (100)	136,338 (100)
**Age (at time of cancer diagnosis)**					
Mean (SD)	76.4 (7)	77 (6.8)	77.6 (6.6)	78.1 (6.3)	78.7 (6.1)
Groupings, N (%)					
66–69	35,686 (19.3)	25,732 (15)	16,513 (10.4)	7,908 (5.4)	0 (0)
70–75	55,931 (30.2)	54,262 (31.5)	52,517 (32.9)	50,734 (34.4)	48,738 (35.8)
76–80	40,937 (22.1)	40,093 (23.3)	39,337 (24.7)	38,571 (26.1)	37,818 (27.7)
81–85	30,894 (16.7)	30,360 (17.7)	29,866 (18.7)	29,409 (19.9)	28,981 (21.3)
85+	21,957 (11.8)	21,595 (12.6)	21,309 (13.4)	21,052 (14.2)	20,801 (15.3)
**Sex**					
Female	94319 (50.9)	88,000 (51.1)	82,089 (51.5)	78,744 (51.4)	70,999 (52.1)
Male	91086 (49.1)	84,042 (48.9)	77,453 (48.5)	74,420 (48.6)	65,339 (47.9)
**Race & ethnicity**					
Non-Hispanic White	156209 (84.3)	145,699 (84.7)	135,665 (85)	126,117 (85.4)	116,812 (85.7)
Non-Hispanic Black	13293 (7.2)	11,872 (6.9)	10,680 (6.7)	9,525 (6.5)	8,572 (6.3)
Hispanic	7363 (4)	6,668 (3.9)	6,036 (3.8)	5,492 (3.7)	4,983 (3.7)
Asian American	7140 (3.9)	6,524 (3.9)	5,988 (3.8)	5,488 (3.7)	5,013 (3.7)
American Indian or Alaskan Native (AIAN)	624 (0.3)	578 (0.3)	528 (0.3)	475 (0.3)	434 (0.3)
Native Hawaiian or another Pacific Islander (NHPI)	470 (0.3)	419 (0.2)	390 (0.2)	354 (0.2)	323 (0.2)
Multiracial	83 (0)	75 (0.05)	72 (0.1)	61 (0.01)	53 (0.04)
Unspecified /Unknown race ^**a**^	223	207	183	162	148
**Marital/ Partner status**					
Yes	64205 (49.5)	59,148 (49.2)	54,473 (48.9)	50,007 (48.5)	45,729 (48.1)
No	65611 (50.5)	61,201 (50.8)	56,981 (51.1)	53,073 (51.5)	49,434 (51.9)
Unknown ^**a**^	55589	51,693	48,088	44,594	41,175
**% Poverty indicator census tract** (Quartiles)					
Highest poverty rate	36,974 (22.1)	34,705 (22.4)	32,559 (22.6)	30,455 (22.8)	28,451 (23.1)
Upper Middle poverty rate	45,147 (27)	42,153 (27.2)	39,243 (27.3)	36,653 (27.5)	34,017 (27.6)
Lower Middle poverty rate	50,906 (30.4)	47,136 (30.4)	43,657 (30.3)	40,328 (30.2)	37,237 (30.2)
Lowest poverty rate	34,184 (20.4)	31,164 (20.1)	28,498 (19.8)	25,919 (19.4)	23,555 (19.1)
Unknown ^**a**^	18,194	16,884	15,585	14,319	13,078
**Neighborhood SES** (US-based Quantile)					
Lowest SES	29,918 (16.8)	27,267 (16.5)	24,936 (16.2)	22,597 (15.9)	20,533 (15.6)
Lower Middle SES	32,006 (17.9)	29,541 (17.8)	27,336 (17.8)	25,113 (17.7)	23,021 (17.5)
Middle SES	33,958 (19)	31,479 (19)	29,190 (19)	27,054 (19)	24,973 (19)
Upper Middle SES	39,000 (21.8)	36,429 (22)	33,826 (22)	31,515 (22.2)	29,272 (22.3)
Highest	34,666 (24.5)	40,950 (24.7)	38,373 (25)	35,932 (25.3)	33,569 (25.6)
Unknown ^**a**^	6857	6376	5881	5,463	4970
**COPD prevalence** Yes No					
	131,230 (70.8)	124,628 (72.5)	117,202 (73.5)	109,429 (74.1)	101,668 (74.6)
	54,175 (29.2)	47,414 (27.5)	42,340 (26.5)	38,245 (25.9)	34,670 (25.4)
**Modified Charlson Comorbidity Score Index** ^**b**^					
0	87434 (47.1)	80,162 (46.6)	73,364 (46)	67,092 (45.4)	64,078 (44.8)
1	43312 (23.4)	40,358 (23.5)	37,635 (23.6)	35,008 (23.7)	34,063 (23.8)
2	24007 (13)	22,606 (13.1)	21,291 (13.3)	19,929 (13.5)	19,564 (13.7)
3+	30652 (16.5)	28,916 (16.8)	27,252 (17.1)	25,645 (17.4)	25,303 (17.7)
**Tumor histology**					
Non-small cell lung cancer	147876 (79.8)	137,054 (79.7)	126,961 (79.6)	117,309 (79.4)	108,121 (79.3)
Small Cell lung cancer	21334 (11.5)	19,486 (11.3)	17,711 (11.1)	16,070 (10.9)	14,544 (10.7)
Other lung cancer	16195 (8.7)	15,502 (9)	14,870 (9.3)	14,295 (9.7)	13,673 (10)
**Early vs. Late Stage**					
Early stage	37431 (21.4)	34,807 (21.5)	29,872 (21.5)	27,943 (21.5)	25,579 (21.6)
Late stage	137764 (78.6)	127,478 (78.5)	108,933 (78.5)	101,817 (78.5)	100,319 (78.4)
Unknown ^**a**^	10210	9,757	8,869	8,517	8,440

^**a**^ Unknown / missing data were not included in calculations of percentage.

^**b**^ Modified CCI: COPD was excluded from CCI to compare comorbidity burden between patients with and without COPD.

### Classification of COPD prevalence and diagnosis timing

**Fig 2:**
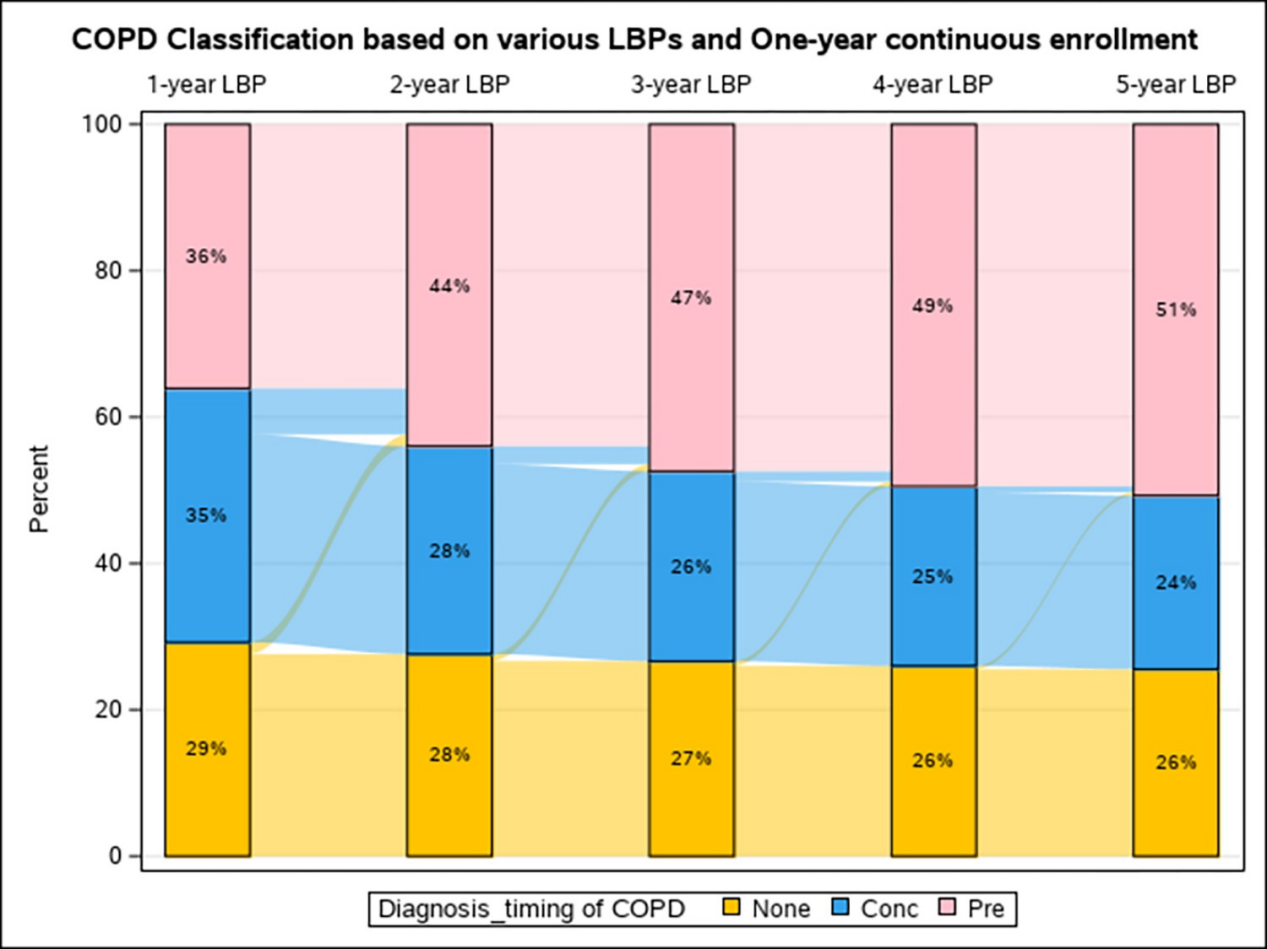
Changes in classification of COPD diagnosis timing by look back period (LBP) among the baseline population with one-year Medicare fee-for -service continuous enrollment (CE). (Method2).

**Fig 3:**
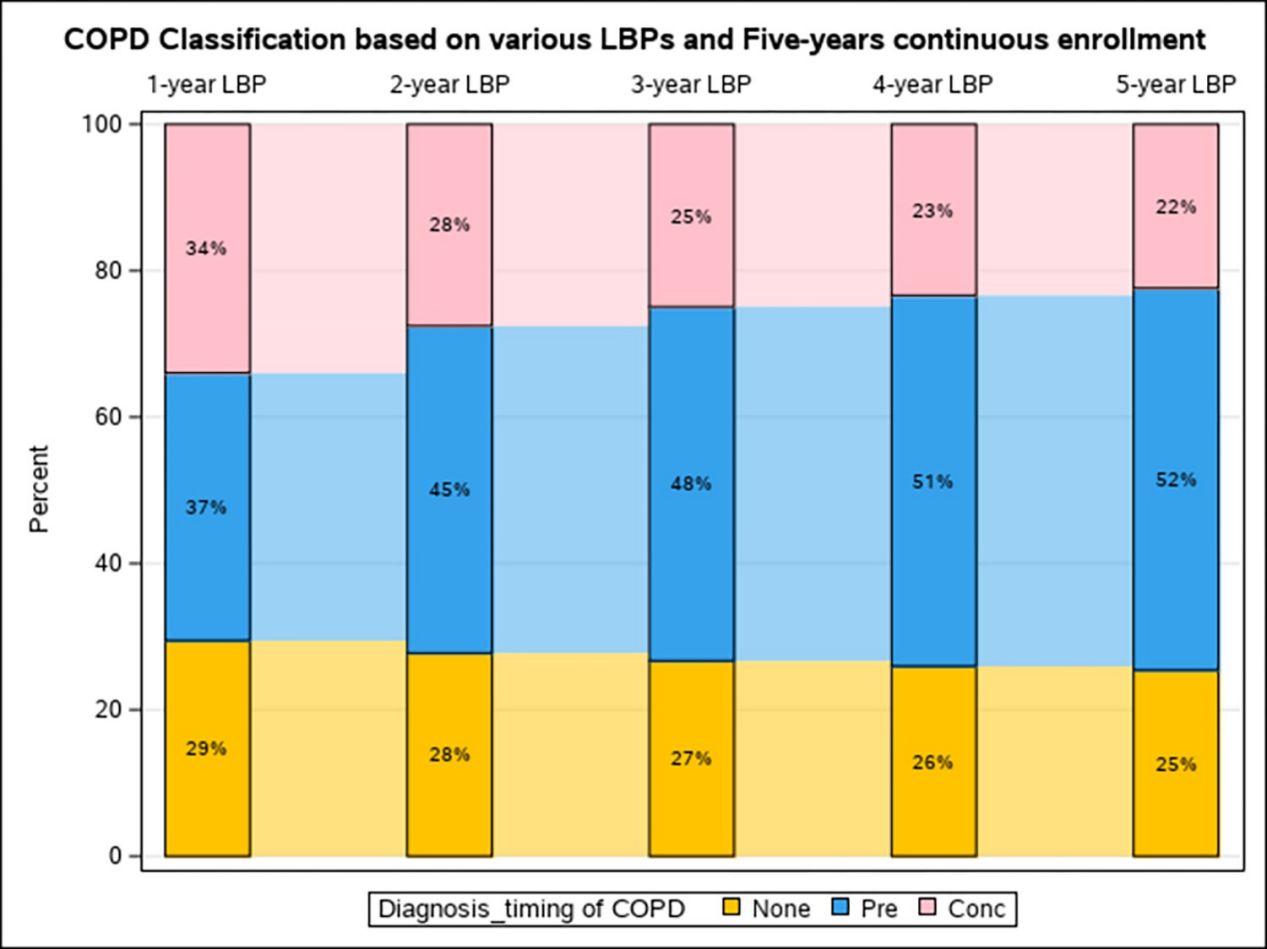
Changes in classification of COPD diagnosis timing by look back period (LBP) among the baseline population with 5-year Medicare fee-for -service continuous enrollment (CE). (Method 3).

**Method 1:** Using a one-year LBP among the baseline population with at least one-year continuous enrollment, we estimated that 36.1% received their first COPD diagnosis at least 3 months before the lung cancer diagnosis (pre-existing COPD), 34.7% within 3 months of lung cancer diagnosis (concurrent COPD), and 29.2% did not have any COPD-related claims (non-COPD. **Method 2:** By extending the LBP to all available LBP (truncated at 5 years) among the baseline population with at least one-year continuous enrollment, we observed gradual increases in pre-existing COPD (from 36.1% to 50.7%), decreases in concurrent COPD (from 34.7% to 23.7%), and decreases in non-COPD (from 29.2% to 25.6%). Notably, as earlier claims were considered (longer LBP), patients who had previously been classified as having concurrent or no COPD were reclassified as having pre-existing COPD as evidence of an earlier diagnosis is discovered. Contrarily, patients with evidence of pre-existing COPD under the one-year LBP classification remained in the pre-existing category no matter how far the LBP was extended (within 5 years). (**Fig 2**) **Method 3:** Despite extending the required continuous enrollment from one-year to at least 5 years, we observed minimal changes in COPD classification, with various LBPs, compared to method 2. The longest LBP of 5 years resulted in ~52% pre-existing COPD, 22.5% concurrent COPD, and 25.5% non-COPD classification, with negligible differences observed when extending the continuous enrollment period beyond one year (**Fig 3 and S1 Table**). **We observed moderate agreement between Methods 1 and 2 with Kappa (95% CI) of** 0.78 (0.77 to 0.78) for simple Kappa testing and 0.67 (0.66 to 0.67) for weighted Kappa testing.

### Sensitivity of COPD classification

Among patients with at least 5 years continuous enrollment, we compared sensitivity of shorter (1, 2, 3, and 4 years) versus longer (5 years) LBPs to classify pre-existing COPD. We observed an increase in sensitivity of pre-existing COPD with longer LBPs, ranging from 70.1% (95% CI: 69.7 to 70.4) for a one-year LBP to 97.1% (95% CI: 97 to 97.2) for a 4-year LBP compared to a 5-year LBP (**Table 2**). In the context of our study design and research question, all available LBP (truncated at 5 years) with a minimum of one-year of continuous enrollment was the best approach to mitigate exposure misclassification of COPD diagnosis timing and losses in sample size. We didn’t apply sensitivity and specificity of the previously validated ICD codes [16,17] in our sensitivity calculations because they were related to validity of ICD codes as diagnostic indicator against pulmonologist chart review. Our measures, however, are related to sensitivity of different observable lookback periods in ascertainment of COPD diagnosis.

**Table 2:** Sensitivity of COPD classification using 1, 2, 3, and 4 year LBPs compared to 5-year LBP among patients with 5 years continuous enrollment (The reference LBP).

Lookback period	Pre-existing COPD N	Sensitivity % of pre-existing COPD (95% CI)
**One year**	49,855	70.1% (69.7 to 70.4)
**Two years**	60,926	85.6% (85.4 to 85.9)
**Three years**	65,935	92.7% (92.5 to 92.9)
**Four years**	69,088	97.1% (97 to 97.2)
**Five years**	**71,141**	100%

## Discussion

In this study, we explored several alternate approaches for searching Medicare claims to identify the best lookback and observability (i.e., continuous enrollment) periods to ascertain diagnosis timing of a chronic condition such as COPD and provide a framework for future studies that would utilize one of these approaches in their methods. Using a large population dataset (SEER-Medicare), we observed that all available LBP with at least one year of continuous enrollment is the most efficient approach to mitigate COPD misclassification while minimizing losses in sample size. With a short observable LBP of one year, there were similar proportions (one third) of patients classified as having pre-existing versus concurrent COPD diagnosis. Extending the LBP increased proportion of pre-exiting COPD from one third to one half of patients.

We observed minimal effect of extending the required continuous enrollment period beyond one year across various LBPs. Prevalence of pre-existing versus concurrent COPD diagnoses were almost the same among those with required one versus 5 year continuous enrollment (Methods 2 versus Method 3) across various LBPs. **Figs 2 and 3** [24,25]. This might be explained by that majority (77.1%) of our study population had at least 5 years of continuous Medicare enrollment, resulting in minimal changes in distribution of sociodemographic (except for age) and clinical characteristics.

Our findings expand upon prior work that considered misclassification of covariates using private and public insurance claims data in the US. A study of the MarketScan database observed increases in prevalence of pre-existing COPD with longer LBP and the negligible effect of extending the required continuous enrollment period [2]. In another study of Medicare claims, researchers compared various fixed LBPs versus all available LBPs to identify and control for baseline covariates. They observed that longer fixed LBPs and all available LBP allowed for more thorough characterization of the study participants and better control of confounding compared to shorter fixed LBPs [3]. The superiority of longer LBPs and all available LBP was also reported by other studies using real world healthcare data [8] and simulation data [26]. In the context of lung cancer, the SEER-Medicare database was the best available option for us to obtain relatively long claims history from older adults in the US. It might not be comparable to the nationwide healthcare databases in other western countries (e.g., Denmark, Sweden) with over a decade claims history. However, even studies from these databases didn’t reach a consensus on the optimum LBP and required observability to identify incident versus prevalent chronic conditions [27–30]. Differences in age distribution, burden of comorbidities, case definition, data source, and study design were among the most identified factors for this lack of consensus.

Unlike the approaches used in this study, some researchers reported that exposure misclassification can be introduced through variability in data elements used to define it rather than variability in claims search period. In a previous study, restricting the analysis to only patients with high quality indicators rather than all patients with common indicators led to a loss in sample size and a biased estimate [6]. In the context of our study, COPD diagnosis is challenging because of inconsistent diagnostic indicators used across different care practices and underutilization of high-quality indicators such as pulmonary function testing (PFTs) [31,32]. Therefore, we used highly performing and externally validated algorithm of ICD codes to ascertain COPD diagnosis to avoid the need for specific test that aren’t frequently done (such as pulmonary function test), and if performed, their results aren’t recorded in claims data.

### Strengths and limitations

While only a methodological exercise, our study utilized a large population-based database that is generally representative of older patients with lung cancer in the US. The longitudinal nature of Medicare data collection allowed ascertainment of COPD diagnosis over a long period (5 years) which improves upon the current recommendations of 1 to 2 years to identify the first diagnosis timing of a chronic condition. However, our study has some limitations: ***first*,** Medicare claims might not be the best option for answering the question of COPD incidence versus prevalence because it is impossible to know with 100% accuracy the first time COPD was diagnosed as no data is available before the usual age of Medicare eligibility at 65 years old. However, evidence from previous studies showed that large proportion (45–90%) of COPD is undiagnosed till the time of lung cancer diagnosis [12,13,33]. ***Second***, we lacked data about COPD severity and smoking which might affect COPD related HCU and diagnosis timing, however these concepts are not easily gleaned from claims data. ***Third***, the sensitivity and specificity of ICD codes we used to ascertain COPD diagnosis might vary compared to the original algorithm, especially around the time of lung cancer diagnosis when it is hard to differentiate if healthcare utilization was related to lung cancer versus underlying COPD. In addition, the age distribution of our study population is older than those used to validate the COPD diagnosis algorithm. Studies that use different COPD diagnostic indicators might have different sensitivity of various LBPs than ours [2]. However, this is an inherent limitation in comparable studies that ascertain chronic condition in the context of cancer diagnosis [15]. ***Fourth***, our study didn’t account for scenario of individuals who were enrolled within 3–12 months (since they would have less than one year of history but enough time for a COPD diagnosis to be establish to count as pre-existing), and how they differ from those who were enrolled in the plan for a longer period of time. ***Fifth***, the scope of this study is not to examine the association of COPD with lung cancer outcomes, but to ascertain prevalent versus incident COPD diagnosis based on documented healthcare utilization in claims data. We encourage researchers who use our methods for association analyses to control for factors that might confound healthcare utilization such as smoking and comorbidity.

## Conclusion

Exposure misclassification of a chronic condition is common using shorter versus longer LBPs in administrative claims data. The length of optimum LBP and continuous enrollment (i.e., observability) period depends on the context of the research question and the data generating mechanisms. In Medicare FFS claims, we estimated that all available LBP with one year of required continuous enrollment was the best approach to tradeoff between COPD diagnosis misclassification and loss in sample size.

## Supporting information

S1 Table Changes in classification of COPD diagnosis timing based on various required continuous enrollment and lookback periods. (DOCX)

S1 Text Method of calculating the 95% CI of sensitivity analyses. (DOCX)

## References

[1] SEER-Medicare. Measures that are limited or not available in the data 2023. Available from: https://healthcaredelivery.cancer.gov/seermedicare/considerations/measures.html#4.

[2] Rassen JA, Bartels DB, Schneeweiss S, Patrick AR, Murk W. Measuring prevalence and incidence of chronic conditions in claims and electronic health record databases. Clin Epidemiol. 2019;11:1–15. Epub 2018/12/28. doi: 10.2147/CLEP.S181242 ; PubMed Central PMCID: PMC6301730. 30588119 PMC6301730

[3] Conover MM, Stürmer T, Poole C, Glynn RJ, Simpson RJ Jr., Pate V, et al. Classifying medical histories in US Medicare beneficiaries using fixed vs all-available look-back approaches. Pharmacoepidemiol Drug Saf. 2018;27(7):771–80. Epub 2018/04/15. doi: 10.1002/pds.4435 ; PubMed Central PMCID: PMC6417795. 29655187 PMC6417795

[4] Lash TL, Mor V, Wieland D, Ferrucci L, Satariano W, Silliman RA. Methodology, design, and analytic techniques to address measurement of comorbid disease. J Gerontol A Biol Sci Med Sci. 2007;62(3):281–5. Epub 2007/03/29. doi: 10.1093/gerona/62.3.281 ; PubMed Central PMCID: PMC2645650. 17389725 PMC2645650

[5] Greenland S. The effect of misclassification in the presence of covariates. Am J Epidemiol. 1980;112(4):564–9. Epub 1980/10/01. doi: 10.1093/oxfordjournals.aje.a113025 . 7424903

[6] Hubbard RA, Lett E, Ho GYF, Chubak J. Characterizing Bias Due to Differential Exposure Ascertainment in Electronic Health Record Data. Health Serv Outcomes Res Methodol. 2021;21(3):309–23. Epub 2021/08/10. doi: 10.1007/s10742-020-00235-3 ; PubMed Central PMCID: PMC8336686. 34366704 PMC8336686

[7] Riis AH, Johansen MB, Jacobsen JB, Brookhart MA, Stürmer T, Støvring H. Short look-back periods in pharmacoepidemiologic studies of new users of antibiotics and asthma medications introduce severe misclassification. Pharmacoepidemiol Drug Saf. 2015;24(5):478–85. Epub 2015/01/21. doi: 10.1002/pds.3738 . 25601142

[8] Nakasian SS, Rassen JA, Franklin JM. Effects of expanding the look-back period to all available data in the assessment of covariates. Pharmacoepidemiol Drug Saf. 2017;26(8):890–9. Epub 2017/04/12. doi: 10.1002/pds.4210 . 28397352

[9] Connolly JG, Schneeweiss S, Glynn RJ, Gagne JJ. Quantifying bias reduction with fixed-duration versus all-available covariate assessment periods. Pharmacoepidemiol Drug Saf. 2019;28(5):665–70. Epub 2019/02/21. doi: 10.1002/pds.4729 . 30786103

[10] Metwally EM, Rivera MP, Durham DD, Lane L, Perera P, Lamb D, et al. Lung Cancer Screening in Individuals With and Without Lung-Related Comorbidities. JAMA Network Open. 2022;5(9):e2230146-e. doi: 10.1001/jamanetworkopen.2022.30146 36066893 PMC9449784

[11] Eman M. Metwally JLL, Bradley Drummond M., Hinton Sharon Peacock, Poole Charles, Thompson Caroline A. COPD with Lung Cancer among Older US Adults: Prevalence, Diagnostic Timeliness, and Association with Earlier Stage Tumors Submitted for publication at the Journal of COPD Foundation 2023.

[12] Ho T, Cusack RP, Chaudhary N, Satia I, Kurmi OP. Under- and over-diagnosis of COPD: a global perspective. Breathe (Sheff). 2019;15(1):24–35. Epub 2019/03/07. doi: 10.1183/20734735.0346-2018 ; PubMed Central PMCID: PMC6395975. 30838057 PMC6395975

[13] Johnson KM, Bryan S, Ghanbarian S, Sin DD, Sadatsafavi M. Characterizing undiagnosed chronic obstructive pulmonary disease: a systematic review and meta-analysis. Respiratory Research. 2018;19(1):26. doi: 10.1186/s12931-018-0731-1 29415723 PMC5803996

[14] Surveillance E, and End Results (SEER) Program (www.seer.cancer.gov) SEER*Stat Database: Incidence—SEER Research Data, 8 Registries, Nov 2021 Sub (1975–2019)—Linked To County Attributes—Time Dependent (1990–2019) Income/Rurality, 1969–2020 Counties, National Cancer Institute, DCCPS, Surveillance Research Program, released April 2022, based on the November 2021 submission.

[15] Maringe C, Fowler H, Rachet B, Luque-Fernandez MA. Reproducibility, reliability and validity of population-based administrative health data for the assessment of cancer non-related comorbidities. PLoS One. 2017;12(3):e0172814. Epub 2017/03/07. doi: 10.1371/journal.pone.0172814 ; PubMed Central PMCID: PMC5338773. 28263996 PMC5338773

[16] Gershon AS, Wang C, Guan J, Vasilevska-Ristovska J, Cicutto L, To T. Identifying individuals with physcian diagnosed COPD in health administrative databases. Copd. 2009;6(5):388–94. Epub 2009/10/30. doi: 10.1080/15412550903140865 . 19863368

[17] Crighton EJ, Ragetlie R, Luo J, To T, Gershon A. A spatial analysis of COPD prevalence, incidence, mortality and health service use in Ontario. Health Rep. 2015;26(3):10–8. Epub 2015/03/19. . 25785665

[18] Yost K, Perkins C, Cohen R, Morris C, Wright W. Socioeconomic status and breast cancer incidence in California for different race/ethnic groups. Cancer Causes & Control. 2001;12(8):703–11. doi: 10.1023/a:1011240019516 11562110

[19] (SEER) TNCIN-SEaERP. Census Tract-level SES and Rurality Database (2006–2018). Available from: https://seer.cancer.gov/seerstat/databases/census-tract/index.html.

[20] Thompson CA, Kurian AW, Luft HS. Linking electronic health records to better understand breast cancer patient pathways within and between two health systems. EGEMS (Wash DC). 2015;3(1):1127. Epub 2015/05/21. doi: 10.13063/2327-9214.1127 ; PubMed Central PMCID: PMC4435001. 25992389 PMC4435001

[21] Enewold L, Parsons H, Zhao L, Bott D, Rivera DR, Barrett MJ, et al. Updated Overview of the SEER-Medicare Data: Enhanced Content and Applications. J Natl Cancer Inst Monogr. 2020;2020(55):3–13. Epub 2020/05/16. doi: 10.1093/jncimonographs/lgz029 ; PubMed Central PMCID: PMC7225666. 32412076 PMC7225666

[22] Matthew Duchnowski ETS. Calculate All Kappa Statistics in One Step. Paper 1825–2014 [Internet]. 2014.

[23] Morton A, Mengersen Kerrie, Whitby Michael, & Playford Geoffrey. Statistical Methods for Hospital Monitoring with R: Statistics in Practice. John Wiley & Sons, Chichester, UK.; 2013.

[24] Otto E, Culakova E, Meng S, Zhang Z, Xu H, Mohile S, et al. Overview of Sankey flow diagrams: Focusing on symptom trajectories in older adults with advanced cancer. Journal of Geriatric Oncology. 2022;13(5):742–6. doi: 10.1016/j.jgo.2021.12.017 35000890 PMC9232856

[25] Shane Rosanbalm R, Inc., Chapel Hill, NC. Getting Sankey with Bar Charts. PharmaSUG 2015—Paper DV072015.

[26] Brunelli SM, Gagne JJ, Huybrechts KF, Wang SV, Patrick AR, Rothman KJ, et al. Estimation using all available covariate information versus a fixed look-back window for dichotomous covariates. Pharmacoepidemiol Drug Saf. 2013;22(5):542–50. Epub 2013/03/26. doi: 10.1002/pds.3434 ; PubMed Central PMCID: PMC3653131. 23526818 PMC3653131

[27] Ødegaard KM, Lirhus SS, Melberg HO, Hallén J, Halvorsen S. A nationwide registry study on heart failure in Norway from 2008 to 2018: variations in lookback period affect incidence estimates. BMC Cardiovasc Disord. 2022;22(1):88. Epub 2022/03/07. doi: 10.1186/s12872-022-02522-y ; PubMed Central PMCID: PMC8898410. 35247979 PMC8898410

[28] Worthington JM, Gattellari M, Goumas C, Jalaludin B. Differentiating Incident from Recurrent Stroke Using Administrative Data: The Impact of Varying Lengths of Look-Back Periods on the Risk of Misclassification. Neuroepidemiology. 2017;48(3–4):111–8. Epub 2017/06/22. doi: 10.1159/000478016 . 28637036

[29] Abbas S, Ihle P, Köster I, Schubert I. Estimation of disease incidence in claims data dependent on the length of follow-up: a methodological approach. Health Serv Res. 2012;47(2):746–55. Epub 2011/10/01. doi: 10.1111/j.1475-6773.2011.01325.x ; PubMed Central PMCID: PMC3419886. 21958217 PMC3419886

[30] Czwikla J, Jobski K, Schink T. The impact of the lookback period and definition of confirmatory events on the identification of incident cancer cases in administrative data. BMC Med Res Methodol. 2017;17(1):122. Epub 2017/08/16. doi: 10.1186/s12874-017-0407-4 ; PubMed Central PMCID: PMC5556662. 28806932 PMC5556662

[31] Ritchie AI, Baker JR, Parekh TM, Allinson JP, Bhatt SP, Donnelly LE, et al. Update in Chronic Obstructive Pulmonary Disease 2020. Am J Respir Crit Care Med. 2021;204(1):14–22. Epub 2021/04/16. doi: 10.1164/rccm.202102-0253UP ; PubMed Central PMCID: PMC8437128. 33856972 PMC8437128

[32] Ragaišienė G, Kibarskytė R, Gauronskaitė R, Giedraitytė M, Dapšauskaitė A, Kasiulevičius V, et al. Diagnosing COPD in primary care: what has real life practice got to do with guidelines? Multidisciplinary Respiratory Medicine. 2019;14(1):28. doi: 10.1186/s40248-019-0191-6 31516702 PMC6732826

[33] Butler SJ, Louie AV, Sutradhar R, Paszat L, Brooks D, Gershon AS. Association between COPD and Stage of Lung Cancer Diagnosis: A Population-Based Study. Curr Oncol. 2023;30(7):6397–410. Epub 2023/07/28. doi: 10.3390/curroncol30070471 ; PubMed Central PMCID: PMC10377848. 37504331 PMC10377848

