# Supplementary material for: Impact of observability period on the classification of COPD diagnosis timing among Medicare beneficiaries with lung cancer
Source: PLOS Digit Health. 2024 Oct 22;3(10):e0000633. doi: 10.1371/journal.pdig.0000633 (PMC11495636; doi:10.1371/journal.pdig.0000633)
Supplement: S1 Table — (DOCX) [file pdig.0000633.s001.docx]

**S1 Table.** **Changes in classification of COPD diagnosis timing based on various required continuous enrollment and lookback periods**.

| **Minimal required continuous enrollment (Observability)** | **Lookback period** | **Pre-existing COPD** | **Concurrent COPD** | **Non-COPD** |
| --- | --- | --- | --- | --- |
| **One year**  **N= 185,405**  **(100% of the baseline population)** | 12 months | 66,867  (36.1%) | 64,363  (34.7%) | 54,175  (29.2%) |
|  | 24 months | 81,520  (44.0%) | 52,677  (28.4%) | 51,208  (27.6%) |
|  | 36 months | 87877  (47.3%) | 48106  (26%) | 49,422  (26.7%) |
|  | 48 months | 91,707  (49.5%) | 45,477  (24.5%) | 48,221  (26%) |
|  | 60 months | 94,034  **(50.7%)** | 43,973  **(23.7%)** | 47398  **(25.6%)** |
| **Two-year**  **N= 172,042**  **(92.8% of the baseline population)** | 12 months | 62,432  (36.3%) | 59,356  (34.5%) | 50,254  (29.2%) |
|  | 24 months | 76,294  (44.4%) | 48,334  (28.1%) | 47,414  (27.5%) |
|  | 36 months | 82,501  (48%) | 43,880  (25.5%) | 45,661  (26.5%) |
|  | 48 months | 86,238  (50.1%) | 41,323  (24%) | 44,481  (25.9%) |
|  | 60 months | 88,495  **(51.4%)** | 39,859  **(23.2%)** | 43,688  **(25.4%)** |
| **Three-year**  **N=159,542**  **(86.1% of the baseline population)** | 12 months | 58,105  (36.4%) | 54,771  (34.3%) | 46,666  (29.3%) |
|  | 24 months | 71,000  (44.5%) | 45,541  (27.9%) | 44,001  (27.6%) |
|  | 36 months | 76,872  (48.2%) | 40,330  (25.3%) | 42,340  (26.5%) |
|  | 48 months | 80,504  (50.5%) | 37,843  (23.7%) | 41,195  (25.8%) |
|  | 60 months | 82,711  **(51.8%)** | 36,416  **(22.8%)** | 40,415  **(25.3%)** |
| **Four-year**  **N=147,674**  (79.7% of the baseline population) | 12 months | 53,878  (36.5%) | 50,426  (34.1%) | 43,370  (29.4%) |
|  | 24 months | 65,868  (44.6%) | 40,929  (27.7%) | 40,877  (27.7%) |
|  | 36 months | 71,309  (48.3%) | 37,028  (25.1%) | 39,337  (26.6%) |
|  | 48 months | 74,740  (50.6%) | 34,689  (23.5%) | 38,245  (25.9%) |
|  | 60 months | 76,905  **(52.1%)** | 33,290  **(22.5%)** | 37,479  **(25.4%)** |
| **Five-year**  **N=136,338**  **(73.5% of the baseline population)** | 12 months | 49,855  (36.6%) | 46,305  (34%) | 40,178  (29.4%) |
|  | 24 months | 60,926  (44.7%) | 37,556  (27.6%) | 37,856  (27.7%) |
|  | 36 months | 65,935  (48.4%) | 33,990  (24.9%) | 36,413  (26.7%) |
|  | 48 months | 69,088  (50.6%) | 31,847  (23.4%) | 35,403  (26%) |
|  | 60 months | 71,141  **(52.2%)** | 30,527  **(22.4%)** | 34,670  **(25.4%)** |
