# Supplementary material for: Impact of observability period on the classification of COPD diagnosis timing among Medicare beneficiaries with lung cancer
Source: PLOS Digit Health. 2024 Oct 22;3(10):e0000633. doi: 10.1371/journal.pdig.0000633 (PMC11495636; doi:10.1371/journal.pdig.0000633)
Supplement: S1 Text — (DOCX) [file pdig.0000633.s002.docx]

**S1 Text. Method of calculating the 95% CI of sensitivity analyses.**

SE = Square root ((Sensitivity * (1- sensitivity)) / Total sample size of patients with at least 5 years of continuous Medicare enrollment)

Upper confidence limit (UCL)= (Sensitivity /100 + Z *SE) * 100.

Lower confidence limit (LCL)= (Sensitivity/ 100 – Z *SE) * 100.

Where Z is the standard normal value corresponding to the confidence level (Z=1.96 for 95% CI).
